# *Arid1a* Loss Enhances Disease Progression in a Murine Model of Osteosarcoma

**DOI:** 10.3390/cancers16152725

**Published:** 2024-07-31

**Authors:** Kaniz Fatema, Yanliang Wang, Adriene Pavek, Zachary Larson, Christopher Nartker, Shawn Plyler, Amanda Jeppesen, Breanna Mehling, Mario R. Capecchi, Kevin B. Jones, Jared J. Barrott

**Affiliations:** 1 Department of Biomedical and Pharmaceutical Sciences, College of Pharmacy, Idaho State University, Pocatello, ID 83209, USA; kanizfatema@isu.edu (K.F.); adriene.pavek@isu.edu (A.P.); nartchri@isu.edu (C.N.); shawn.plyler@commonspirit.org (S.P.); amandajeppesen@isu.edu (A.J.); breannamehling@isu.edu (B.M.); 2 Department of Oncological Sciences, University of Utah School of Medicine, Salt Lake City, UT 84132, USA; wangyangliang@hotmail.com; 3 Department of Orthopaedics, University of Utah School of Medicine, Salt Lake City, UT 84132, USA; 4 Huntsman Cancer Institute, Salt Lake City, UT 84112, USA; 5 Department of Human Genetics, University of Utah School of Medicine, Salt Lake City, UT 84132, USA; mario.capecchi@genetics.utah.edu; 6 Department of Cell Biology and Physiology, Brigham Young University, Provo, UT 84602, USA; 7 Simmons Center for Cancer Research, Provo, UT 84602, USA

**Keywords:** osteosarcoma, *Arid1a*, SWI/SNF, *piggyBac*, genomic instability, epigenetics, forward genetic screen, DNA damage repair, DSB, SSB

## Abstract

**Simple Summary:**

Osteosarcoma is an aggressive form of bone cancer that spreads quickly and is challenging to treat. Genetic screening in this study discovered that the loss of the *Arid1a* gene is a crucial factor in the development and progression of osteosarcoma. In vitro and in vivo studies validated that the loss of *Arid1a* increased proliferation and chemoresistance in human cell lines and led to higher disease penetrance, metastases, and shorter survival in mice. Unbiased pathway analyses suggest that *Arid1a* may contribute to the aggressiveness of osteosarcoma by dysregulating genomic instability.

**Abstract:**

Osteosarcoma is an aggressive bone malignancy, molecularly characterized by acquired genome complexity and frequent loss of *TP53* and *RB1*. Obtaining a molecular understanding of the initiating mutations of osteosarcomagenesis has been challenged by the difficulty of parsing between passenger and driver mutations in genes. Here, a forward genetic screen in a genetic mouse model of osteosarcomagenesis initiated by *Trp53* and *Rb1* conditional loss in pre-osteoblasts identified that *Arid1a* loss contributes to OS progression. *Arid1a* is a member of the canonical BAF (SWI/SNF) complex and a known tumor suppressor gene in other cancers. We hypothesized that the loss of *Arid1a* increases the rate of tumor progression and metastasis. Phenotypic evaluation upon in vitro and in vivo deletion of *Arid1a* validated this hypothesis. Gene expression and pathway analysis revealed a correlation between *Arid1a* loss and genomic instability, and the subsequent dysregulation of genes involved in DNA DSB or SSB repair pathways. The most significant of these transcriptional changes was a concomitant decrease in *DCLRE1C*. Our findings suggest that *Arid1a* plays a role in genomic instability in aggressive osteosarcoma and a better understanding of this correlation can help with clinical prognoses and personalized patient care.

## 1. Introduction

Osteosarcoma (OS) is a primary bone malignancy and a leading cause of cancer-related mortality among children and young adults in the USA [1,2,3]. Patients with metastasis have a dismal prognosis with 5-year survival rates ranging from 23 to 29% [2,4,5]. Recent sequencing technologies have identified numerous gene mutations and pathways associated with OS pathogenesis [6,7]. Identifying a consistent molecular event linked to osteosarcomagenesis and its progression has been challenging because of the extensive mutational landscape and intratumoral and intertumoral heterogeneity. Osteosarcoma is a genomically complex cancer [8,9] characterized by significant chromosomal rearrangements and genomic instability (GI). Both genetic and epigenetic mechanisms contribute to GI in OS [8,9,10]. The most consistent mutations found in OS are loss-of-function mutations in *TP53* (mutation frequency from 16% to 90%) and *RB1* (mutation frequency from 10% to 64%) [6].

It is, therefore, critical to develop a model system that best recapitulates genetic heterogeneity and identifies driver mutations. Insertional mutagenesis has been widely used for forward genetic screenings to detect novel cancer genes [11]. Prospective modeling in OS demonstrated that *Trp53* and *Rb1* gene disruption in osteoblasts are dominant events; however, they are not sufficient to induce tumor formation [12,13]. Even with both genes disrupted in mice pre-osteoblasts, the penetrance of tumor formation was less than 70%, and when it does occur, typically only one or two tumors per mouse arise [12]. This suggests that additional disruptions are probably contributing to osteosarcomagenesis. However, due to a substantial number of genetic and epigenetic anomalies observed in human OS cells [14], it has been challenging to determine which of these are critical for the progression of OS versus passengers to it. Previously, Moriarty et al. performed a forward genetic screen in mice using *Sleeping Beauty* transposons with and without somatic activation of a dominant negative *Trp53* allele to identify genes driving OS [15]. They found 232 transposon integration sites associated with OS development and 43 sites associated with metastasis [15].

In this study, we utilized a single insertion transposon, *piggyBac*, with a background of both *Trp53* and *Rb1* loss. One of the most frequent insertions identified disrupted the gene *Arid1a* (AT-rich interactive domain-containing protein 1 A). *Arid1a* is the most frequently mutated subunit of the canonical BAF (BRG1/BRM and Associated Factors) chromatin remodeling complex (mammalian homolog of SWI/SNF) [16,17]. Inactivating mutations in this gene have been found in a broad range of human cancers bearing mutation prevalence ranging from 29% to 60% [18,19,20,21]. However, the role of *Arid1a* mutations in human cancers is still a topic of debate. While several studies have linked *Arid1a* loss to a worse prognosis in various cancers, such as ovarian, endometrial, colon, and gastric cancer, some studies suggest that the overexpression of *Arid1a* may also play a role in oncogenesis [20,22,23,24,25,26]. This indicates that *Arid1a* plays a context- and tissue-dependent role in carcinogenesis.

In OS, *Arid1a* has been found to exhibit a tumor-suppressive function both in cell lines and human samples, where its loss associates with a worse prognosis [21,27]. Our in vitro experiments confirmed these findings that *Arid1a* loss resulted in a more proliferative and chemoresistant phenotype. Similarly, our murine osteosarcomagenesis experiments revealed that *Arid1a* loss prompts rapid tumorigenesis and more consistent progression to metastasis. Our transcriptional analysis demonstrated that *Arid1a* loss significantly dysregulated the expression of genes that are essential for maintaining genome stability pathways such as non-homologous end-joining (NHEJ), homologous recombination (HR), and nuclear excision repair (NER) pathways. As an integral member of the cBAF complex, *Arid1a* plays a critical role in maintaining genomic integrity by regulating DNA damage repair pathways [23,28,29,30]. Consequently, the loss of *Arid1a* leads to significant dysregulation in GI observed in various cancers, including OS [18,24,28,30,31]. GI, characterized by frequent mutations and chromosomal aberrations, is one of fourteen hallmarks of cancer and portends a worse prognosis, depending on the cancer context [32,33,34,35,36]. Several studies have established an association between dysfunctional DDR pathways in cancers with *Arid1a* mutations [18,37]. We hypothesize that *Arid1a* plays a critical role in aggressive OS by manipulating genome stability pathways. As such, it is important to further investigate this phenomenon to gain a better understanding of its implications.

## 2. Materials and Methods

### 2.1. Transposon Constructs for Forward Genetic Screening

The cDNA for the mouse codon-optimized piggyBac transposase, along with the sequence for the ERT2 domain, were obtained from Dr. Sen Wu. Previous evaluations have demonstrated its efficacy in controlling piggyBac transposase activity in vitro. The two unique transposable elements have been designed as modifications to the existing *piggyBac* transposable elements available in the laboratory. These modifications incorporate key features from the *T2/Onc3* transposable elements used in conjunction with *Sleeping Beauty*, as provided by our collaborator, Adam Dupuy. Each of the modified elements was included as an epitope tag downstream of the splice acceptor site. In *PBonc1*, the epitope tag was the c-Myc epitope. Both codons were optimized for the mouse and positioned consecutively in all three reading frames. This arrangement ensured that each frame terminated with the epitope tag, immediately followed by a stop codon, facilitating the shift to the next frame’s epitope tag. In *PBonc2*, the epitope tag used was hemagglutinin (HA), similarly positioned in all three possible reading frames. Additionally, each transposable element was equipped with unique sequencing primer sites located near its ends (Supplemental Figure S1a).

### 2.2. Animal Model Used in the piggyBac Mutagenesis System

Breeding mouse colonies with conditional alleles of *Rb1* and *Trp53* were established, and these two strains were crossed to breed homozygous alleles for both. Genotyping for the floxed alleles was performed by PCR with the tail or ear tissue-derived DNA following IACUC-approved procurement protocols. In total, 38 mice were generated that were homozygous for floxed *Trp53* and *Rb1* in the presence of the *Osterix-CreERT2* transgene and heterozygous for a novel piggyBacERT2 knocked into the *Rosa26* locus. All mice received tamoxifen via IP injection at the time of weaning and one week later to ensure activation of the *piggyBac* transposase after its expression had been enabled by tissue-specific, tamoxifen-inducible Cre-mediated excision of the floxed stop cassette preceding it. A total of 88 tumors were harvested from 38 mice, and each skeletal tumor was divided into two specimens, the first prepared for histologic analysis and the second for genetic analysis. The first tumor specimen intended for histologic analysis was fixed, decalcified, sectioned, and stained with H&E for histopathologic confirmation of the diagnosis.

### 2.3. Splinkerette PCR and CIS Analysis

The method of splinkerette PCR was applied to locate the insertional mutation in the mouse genome, following the published protocol [38]. Following restriction enzyme Sau3a1 digestion of tumor genomic DNA, the *PBonc* transposon IR/DR nearby was cut into fragments with its neighboring region in genomic DNA with an overhang of “gatc”. The fragments were then ligated on both ends with the splinkerette adaptor pairs, incorporating a hairpin structure.

Following the splinkerette adaptor ligation, fragments were digested with the restriction enzyme EcoRV to eliminate the splinkerette adaptor ligated with the transposon end. The first round of PCR was performed with one primer recognizing the splinkerette adaptor long strand and another primer recognizing the left part of the transposon IR/DR sequence (forward primer: PB-N Splink 1: 5′-AGTAACCGTTGCTAGGAGAGA-3′; reverse primer: PB-R YW-SP-1: 5′-CGTCAATTTTACGCATGATTATC-3′). The second round was performed as nested PCR with the splinkerette-PCR primer incorporated with specifically designed ‘barcode’ sequences corresponding to different samples. Ion Torrent DNA sequencing was adopted for the sequencing of PCR products (A adapter with barcode: 5′-CCATCTCATCCCTGCGTGTCTCCGACTCAGXXXXXXXXXXGCTGAATGAGACTGGTGT, P1 adapter: CCACTACGCCTCCGCTTTCCTCTCTATGGGCAGTCGGT-GATAACGTACGTCACAATATGAT). The transposon of *PBonc* insertion sites in the genome was determined by BLAST with the sample-specific sequencing data and the CIS among different samples were analyzed to detect the most frequent CIS in the genome.

### 2.4. Human OS Cell Lines and Cell Culture

The two osteosarcoma cell lines (SJSA-1 and U2OS) used in this study were purchased from the American Type Culture Collection biobank (ATCC; Rockville, MD, USA). Cells were cultured and maintained in our lab at 37 °C in a humidified 5% CO_2_ incubator, with culture medium, RPMI 1640 (Caisson Labs, Smithfield, UT, USA) supplemented with 10% FBS (Atlanta biologicals, Flowery Branch, GA, USA) and with 1% penicillin and streptomycin (Atlanta biologicals, Flowery Branch, GA, USA). Cells were split using trypsin-EDTA 1:3 to 1:10, depending on their growth rate, two to three times per week.

### 2.5. Arid1a CRISPR/cas9 Knockout

*Arid1a* knockout was performed via the CRISPR/Cas9 technique using specific sgRNA targeting *Arid1a* in U2OS and SJSA-1 cells. The control CRISPR/Cas9 and GFP fusion protein expression vector (sc-418922; Santa Cruz Biotechnology, Dallas, TX, USA) was used for cloning. The transformation was performed following the One-Shot MAX Efficiency DH5α-T1R Competent Cells (Invitrogen, Waltham, MA, USA, 12297-016) protocol. Transformed plasmids were then isolated and purified using ZymoPURE—Express Plasmid Midiprep Kit (Zymo Research, Tustin, CA, USA) following the manufacturer’s protocol. Purified plasmid DNA concentrations were determined by Qubit 3.0 fluorometer (Invitrogen, Waltham, MA, USA).

The third coding exon of *Arid1a* was selected for guide RNA design; the sgRNA used was GCCTGCTGGGAGAGCGTCGA (Integrated DNA Technologies, Coralville, IA, USA). Transfections were performed with jetPRIME transfection reagent (Polyplus, Illkirch, France) following the manufacturer’s protocol. Briefly, cells were seeded in a six-well plate at the quantity of 1 × 10^5^ cells per 2 mL of serum-containing medium (optimized following jetPRIME protocol). When the cells reached 80–90% confluence, they were transfected with different concentrations of control/Cas9 and Cas9/GFP plasmids (2 μg DNA/well) as per the manufacturer’s instructions and incubated for 24–48 h. Cells were washed with PBS and replaced with serum-containing medium. Transfection efficiency was measured by fluorescence microscope (EVOS-FL; Thermo Fisher Scientific, Waltham, MA, USA), and the wells with higher GFP expressed cells were transfected with sgRNA and incubated. In both cases, cells were starved of the medium for better transfection efficiency. *Arid1a* knockout efficacy was measured by RT-PCR in triplicates.

### 2.6. RT-qPCR

Reverse transcriptase quantitative polymerase chain reaction (RT-PCR) was performed on samples obtained from both metastatic and non-metastatic models. RNA was extracted from samples using a Quick-RNA MiniPrep Plus kit (Zymo, Dustin, CA, USA, R1058) and cDNA was made using Qscript cDNA Supermix (Quantabio, Beverly, MA, USA, 95048). The primers used were as follows: (human) forward—GCAGGGATATCTTACCTGCG, reverse—GGCTCAGTCTCCTTACCAGC; (mouse) forward—TGGGCAAGATGAGACCTCAG, reverse—TCTGCTGTGCATAAGAGAGGC. cDNA was amplified and measured using the Eppendorf Mastercycler Realplex2 (Eppendorf, Hamburg, Germany). Data were analyzed using a delta CT calculation.

### 2.7. Animal Models Used for Conditional Deletion of Arid1a

All procedures were approved by the Idaho State University Institutional Animal Care and Use Committee protocol 757 and 775. Mice bearing LoxP-flanked conditional alleles of *Rb1*, *Trp53*, and *Arid1a* were obtained from Jackson Laboratories. *OsxCreERT2* animals were previously described [39,40,41]. All animals were genotyped using published protocols. Tamoxifen was administered at 1 month of age by injecting a solution of tamoxifen at a concentration of 20 mg/mL intraperitoneally at a dose of 400 μg of tamoxifen per gram of mouse.

### 2.8. Real-Time Growth/Proliferation Assay

First, 1 × 10^5^ cells/mL of SJSA-1 and U2-OS cells were stained with NucBlue Live Ready Probes reagent (Thermo Fisher, Scientific, Waltham, MA, USA, R37605) (2 drops/mL of medium) and plated in a 96-well plate. After that, cells were incubated in the Image Express Pico set for live-cell counting (10× objective) to take images at one-hour intervals for 48 h. During image acquisition, automated image analysis (cell number, individual cell area, and area covered by cells) was performed. Dead cells were identified based on size and shape and excluded from the analysis of healthy cells.

### 2.9. Scratch Migration Assay

SJSA-1 and U2-OS cells were seeded in a 96-well plate at a concentration of 1 × 10^5^ cells/mL and incubated until they reached >90% confluence. We made a scratch using a multichannel pipette (300 μL) and washed the detached cells away using 1X PBS. Immediately after the PBS wash, we re-fed the cells with the medium containing the staining solutions. We left them to grow in the Image Express Pico set for live-cell counting to take images at one-hour intervals for 24 h. The cells were stained with a 100 μL solution of 1 μM CellTrace Calcein green (Thermo Fisher Scientific, Waltham, MA, USA, C348520) and NucBlue Live ReadyProbes (Thermo Fisher Scientific, Waltham, MA, USA, R37605) reagent. Wound areas were measured from the acquired images using the ImageJ wound healing assay plugin [42]. Wound healing assay tools detect the wounded area (region of interest) and quantify total area, scratch width, wound closure (percent), and standard deviation of scratch width (in pixels). Briefly, size-selected images were analyzed under the same variance and threshold values across all the images.

### 2.10. MTT Assay

The MTT (3-[4,5-dimethylthiazol-2-yl]-2,5-diphenyltetrazolium bromide) assay was performed to determine the effects of *Arid1a* loss on chemosensitivity in the osteosarcoma cell lines (U2OS and SJSA-1). Briefly, replicates of both the cell lines (control and knockouts) were seeded into 96-well plates at a density of 10,000 cells/well (in 100 μL RPMI medium). After 24 h of incubation, the cells were treated with different concentrations of doxorubicin for 3 days. Afterward, 10 μL of MTT (5 mg/mL; Sigma-Aldrich, St. Louis, MO, USA) was added to each well, and the cells were incubated for 2.5 h (at 37 °C in a humidified 5% CO_2_ atmosphere). Finally, the resulting formazan product was dissolved with 100 μL of MTT solubilization buffer/acid isopropanol and the absorbance was read at a wavelength of 570 nm on the Varioskan LUX multimode microplate reader (Thermo Fisher Scientific, Waltham, MA, USA, VL0000D0) for each plate. The dose–response curves were fitted using GraphPad Prism (GraphPad Software, La Jolla, CA, USA, version Prism 9.4.1).

### 2.11. Immunohistochemistry and Histology

Mouse tissues were fixed in 4% paraformaldehyde overnight and embedded in paraffin. Paraffin-embedded tissues were stained by immunohistochemistry by rehydrating slides through a Citrisolv and ethanol dilution wash. Hematoxylin and eosin staining were performed as previously described [43].

### 2.12. Imaging Techniques

The EVOS Fluorescence Microscope (Thermo Fisher Scientific, Waltham, MA, USA, AMF4300s) was used to acquire high-resolution images at various magnifications for our in vitro assays. Micro-CT imaging has been performed using the nanoScan SPECT/CT system to generate high-resolution whole-body images of mice at the Center for Quantitative Cancer Imaging (CQCI) at Huntsman Cancer Institute (HCI). Acquired images have been further processed to investigate tumorigenesis using ImageJ (Fiji) software (version 1.54c).

### 2.13. Transcriptome Analysis

Total RNA was isolated with the RNeasy mini kit (QIAGEN, Germantown, MD, USA). For transcriptome sequencing, RNA was prepared using the Illumina TruSeq RNA kit (Illumina, San Diego, CA, USA), checked with the Bioanalyzer RNA 6000 chip (Agilent Technologies, Santa Clara, CA, USA), captured using the RiboZero method (Illumina, San Diego, CA, USA), and 50-cycle end-read sequenced on an Illumina HiSeq 2000. Reference FASTA files were generated by combining the chromosome sequences from mm10 with splice junction sequences generated by USeq (v8.8.8) MakeTranscriptome using Ensembl transcript models (build 74). Reads were aligned with Novoalign (v2.08.01), allowing up to 50 alignments per read. USeq’s SamTranscriptomeParser was used to select the best alignment for each, and the coordinates of reads aligning to splices were converted back to genomic space. Differential gene expression was measured using USeq Defined Region Differential Seq, which counts the number of reads aligned to each gene and then calls DESeq2 (v1.26.0) using default settings. The Ingenuity Pathway Analysis (IPA) suite (QIAGEN, Germantown, MD, USA) was used to identify pathways associated with gene clusters.

### 2.14. Statistics and Analysis

Most statistical analyses were performed using GraphPad Prism 9.4.1 (GraphPad Software, La Jolla, CA, USA). Alternatively, programs within Microsoft Excel were used to analyze and generate graphical data. ANOVA was used before making individual comparisons among a group of samples with different experimental conditions. Two-tailed t-tests were used to compare means of samples and a threshold for significance was set at *p*-value < 0.05. Survival analyses were performed using Kaplan–Meier survival analysis and the log-rank test was utilized to compare survival curves between groups for statistical significance. A comparison of the percentages of metastasis and non-metastasis was performed by Fisher’s exact test with a 2 × 2 contingency table of metastasis and non-metastasis and *Arid1a* KO and *Arid1a* WT. To evaluate the statistical significance of the chemosensitivity in the MTT assay, we performed an ANOVA combined with linear regression called an ANCOVA. This allowed us to examine the influence of the concentration of doxorubicin on cell viability while removing the effect of changing the drug concentration. To compensate for multiple hypothesis testing with our genomics data, we used FDR/Benjamini–Hochberg corrections to calculate the adjusted *p*-value.

## 3. Results

### 3.1. Forward Genetic Screen Identifies Genetic Players of Osteosarcomagenesis

The *piggyBac* transposon-mediated insertional mutagenesis was employed to delineate the critical mutations necessary for the transformation of becoming metastatic that is observed in some human OS. The piggyBac design included bidirectional splice acceptors followed by triple-frame epitope tags and poly-adenylation signals as well as a strong promoter and splice donor site in the middle of each that allow for the discovery of oncogenes and tumor suppressors (Supplemental Figure S1a). In vitro induction of harvested mouse fibroblast cells demonstrated the combinatorial activation of *piggyBac* via the presence of luciferase activity (Supplemental Figure S1b).

**Figure 1:**
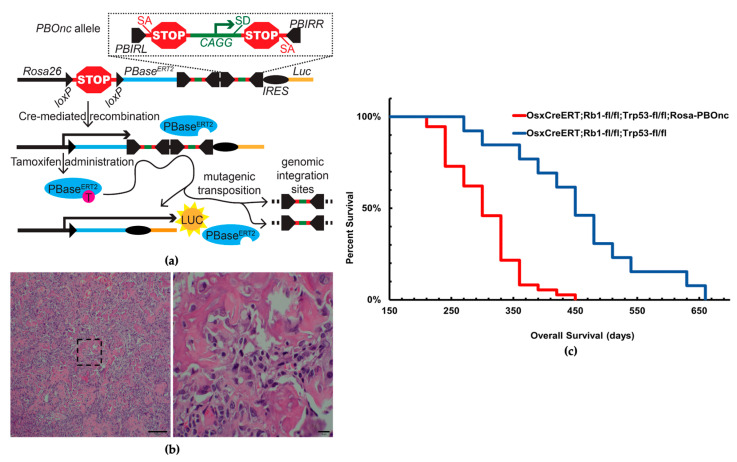
Forward genetic screen using *piggyBac* transposon identifies *Arid1a* as a tumor suppressor gene. (**a**) Schematic of *PBonc* design, random genomic integration of transposable elements from *Rosa26* locus via Cre-lox mediated recombination (PBIRL/PBIRR = piggyBac Inverted Repeat sequences left/right, SA/SD = Splice Acceptor/Donor, PBase = piggyBac transposase, IRES = Internal Ribosomal Entry Sites, Luc = Luciferase). (**b**) Histology of tamoxifen-induced skeletal tumors harvested from *piggyBac* inserted cohort shows similar appearance with osteosarcoma (osteoid matrix = bright pink, scale bar 100 and 10 μm). (**c**) Kaplan–Meier survival analysis of mice with (red) *piggyBac* mutagenesis showed a 5-month survival disadvantage compared to the ones without (blue); median overall survival was 10.0 months and 15.0 months, respectively (*p*-value < 0.0001).

As noted earlier, the most common genetic mutations associated with human OS are loss-of-function mutations in *TP53* and *RB1*. Littermate cohorts of mice—all homozygous for *Trp53^fl/fl^*, *Rb1^fl/fl^*, and bearing the *Osterix-Cre^ERT2^* transgene—were either heterozygous for the novel *PBOnc* allele knocked into the *Rosa26* locus, or wildtype at that locus. All mice received intraperitoneal (IP) injections of tamoxifen (400 μg/g) at the time of weaning and one week later. Cre-mediated, tissue-specific, tamoxifen-inducible excision of the floxed stop cassette prompted conditional inactivation of both alleles of *Trp53* and *Rb1* as well as the expression of the transposase (Figure 1a).

The second administration of tamoxifen ensured nuclear localization and activity of the *piggyBac^ERT2^* transposase, which moved the two transposable elements to traceable locations. In total, 88 tumors from these cohorts were harvested for further histopathologic and genetic analysis. H&E histopathologic staining analysis of the cross-sections of tumors from both groups confirmed osteosarcoma-like appearances (Figure 1b).

The Kaplan–Meier survival study revealed that the mice with the mutagenic insertion of *piggyBac* showed a 5-month survival disadvantage compared to the *Trp53^fl/fl^; Rb1^fl/fl^; OsxCre^ERT2^* mouse cohort (*p*-value < 0.0001) (Figure 1c).

Splinkerette PCR analysis with Ion Torrent DNA sequencing was used following a published protocol for the detection of transposon insertion sites in the mouse genome [38]. The various transposon insertion sites were analyzed to detect the most frequent across the genome. *Arid1a* was among the top genes disrupted by transposon insertion (Supplemental Table S1) and was selected for additional pursuit as a potential contributor to osteosarcomagenesis.

### 3.2. Arid1a Loss Enhances Osteosarcomagenesis in Mice Leading to Poor Survival and a Higher Rate of Metastases

To validate the *piggyBac* screen and examine the impact of *Arid1a* loss on osteosarcomagenesis, we engineered murine models of OS with and without conditional loss of *Arid1a*. Mice with the baseline mutation of *Trp53^fl/fl^; Rb1^fl/fl^; OsxCre^ERT2+/−^* were crossed with *Arid1a^fl/fl^* mice (obtained from Jackson Labs) to generate three different littermate-controlled cohorts of wildtype (*Arid1a^wt/wt^*), homozygous (*Arid1a^fl/fl^*), and heterozygous (*Arid1a^fl/wt^*) alleles. The genotypes were confirmed using PCR to test for the insertion of the floxed allele. After confirming the correct inheritance of the *OsxCre^ERT2^* transgene, mice at the age of 4 weeks received 400 μg/g IP injection of tamoxifen to initiate Cre-mediated recombination and were monitored for any apparent tumorigenesis. Mice survival and overall tumor and metastatic burden were evaluated as a measure of an aggressive phenotype. Any morbid mice were humanely euthanized for further evaluation of primary and metastatic tumors.

**Figure 2:**
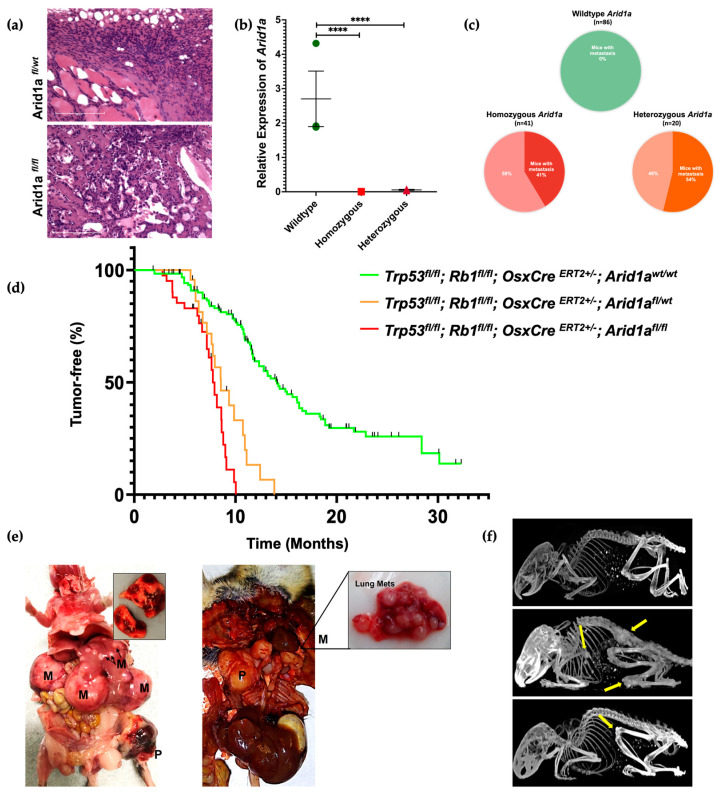
In vivo *Arid1a* knockout resulted in a greater tumor burden and metastasis. (**a**) H&E staining of *Arid1a* homozygous (**bottom**) and heterozygous (**top**) mouse tumor tissue shows an osteosarcoma-like appearance (osteoid matrix = bright pink, scale bar 100 μm). (**b**) Relative expression of *Arid1a* in wildtype, *Arid1a^fl/fl^* and *Arid1a^fl/wt^* mouse tumor samples (**** *p*-value < 0.0001). (**c**) Pie chart shows the percent metastasis found in *Arid1a* in wildtype, *Arid1a^fl/fl^* and *Arid1a^fl/wt^* mice. (**d**) Kaplan–Meier tumor-free curve of *Arid1a* in wildtype (n = 142), *Arid1a^fl/fl^* (n = 41), and *Arid1a^fl/wt^* (n = 22), mice (*p*-value < 0.0001). Black lines represent all the mice that were censored (no tumor). (**e**) Gross necropsy showing the common sites of primary and metastasized tumor formation in *Arid1afl/fl* and *Arid1a^fl/wt^* mice (P = Primary, M = Metastatic tumors). (**f**) Micro-CT image showing the formation of multiple tumors (yellow arrows) on *Arid1a^fl/fl^* mice at various ages—(**top**) *Arid1a* wildtype control, (**middle**) *Arid1a^fl/fl^* mice at 217 days, (**bottom**) *Arid1a^fl/fl^* mice at 91 days.

Phenotypic evaluation of osteosarcomagenesis revealed a 61% penetrance in the mice with the *Arid1a^wt/wt^* allele, while it was 100% in the homozygous and 91% in the heterozygous knockout mice. We analyzed a total of 142 wildtypes, 41 homozygous, and 22 heterozygous mice. Upon histological evaluation of the resected tumors from mice with homozygous and heterozygous loss of *Arid1a*, there was classical evidence of osteosarcoma with the presence of an osteoid matrix. Most samples exhibited hypercellularity containing spindle cell tumor cells with classic pleomorphic nuclei (Figure 2a).

To confirm the absence of *Arid1a* expression in the *Arid1a^fl/fl^* mice, reverse transcription PCR was performed on extracted RNA samples from tumors. There was a significant decrease in the expression level of *Arid1a* in the *Arid1a^fl/fl^ and Arid1a^fl/wt^* mouse cohorts (Figure 2b).

The proportion of metastatic tumors in the *Arid1a* wildtype mice with a primary tumor was 0% (n = 86); for the *Arid1a* homozygous KO mice, it was 41% (n = 41); and for the heterozygous KO mice, it was 55% (n = 20). Fisher’s exact test evaluated the statistical significance of metastatic burden between *Arid1a* wildtype and combined knockout cohorts (*p*-value < 0.00001) (Figure 2c).

To evaluate mice survival, we performed Kaplan–Meier survival analysis on *Arid1a* wildtype (n = 142), *Arid1a^fl/fl^* (n = 41), and *Arid1a^fl/wt^* (n = 22) mice. A significant decrease in survival was observed in mice with either a heterozygous or homozygous loss of *Arid1a*. The median overall survival in the *Arid1a^fl/fl^*, *Arid1a^fl/wt^*, and *Arid1a^wt/wt^* mice was 7.8 months, 8.6 months, and 14.1 months, respectively *(p*-value < 0.0001) (Figure 2d).

Micro-CT imaging, along with gross necropsy, identified that the mice with heterozygous or homozygous deletion of *Arid1a* developed significantly higher numbers of tumors per mouse than the wildtype cohort. The common sites of tumorigenesis for the primary tumors were the spinal region, shoulders, ribs, hindlimbs, base of the tail, and jaws, and for the metastasized tumors, the liver and lungs (Figure 2e,f).

In the cohort of mice that were homozygous for loss of *Arid1a*, we observed 17 mice that died at a very young age (<3 months). Upon gross dissection, no tumors were found. We censored these mice, and they were not included in the survival analysis or the penetrance data. However, we suspect that microlesions in the spinal cord may have led to premature death or the need to be euthanized due to observations of hemiplegia in some mice. CT scans were performed on several younger mice to evaluate the presence of micro spinal lesions (Figure 2e); however, none were found in the small cohort analyzed by CT.

### 3.3. Transcriptomic Analysis Reveals Arid1a Loss Leads to a Genomically More Unstable Osteosarcoma Phenotype

**Figure 3:**
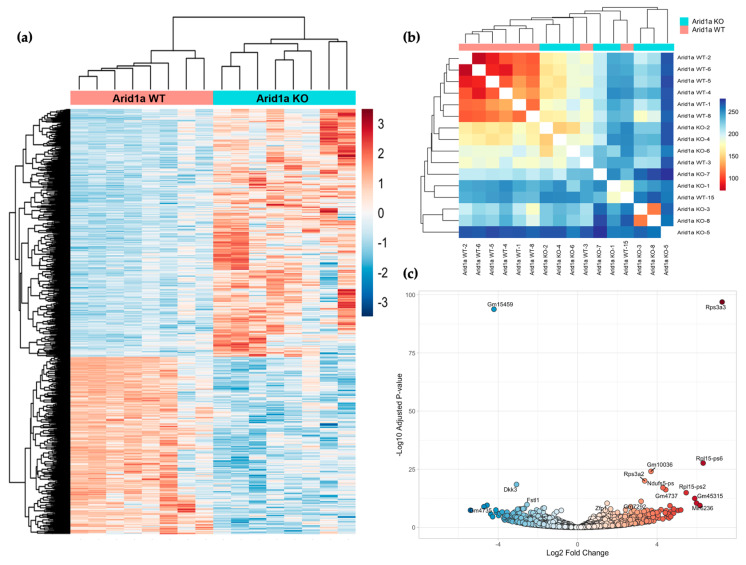
Transcriptional comparison of *Arid1a* WT and *Arid1a* KO osteosarcomas. (**a**) Heatmap demonstrating the distribution of top 1000 significant differentially expressed genes (clustered by Z-scores) between *Arid1a* WT (Red) and *Arid1a* KO (Blue) mouse osteosarcomas. (**b**) Euclidean distance between samples. (**c**) Volcano plot representing most significant DE genes in both cohorts, X-axis representing log_2_ FC and Y-axis presenting the –log_10_ of the adjusted *p*-value.

To investigate the impact of *Arid1a* loss on the osteosarcoma transcriptome, we conducted RNA-sequencing on 16 tumors derived from the mouse models, consisting of eight *Arid1a^fl/fl^* knockout (KO) and eight wildtype (WT) samples. Differential gene expression analysis revealed 2154 genes exhibiting differential expression between the two cohorts, with 1320 genes upregulated and 834 genes downregulated (Figure 3a). Hierarchical cluster analysis demonstrated that the *Arid1a* WT tumors formed a tightly clustered group, while the KO tumors displayed more significant heterogeneity. There were two *Arid1a* WT mice with tumors with similar downregulated genes as other WT tumors; however, the upregulated genes normally seen in the WT tumors were absent, thus resembling the KO tumors and hence the intermixing of these two samples with the KO samples (Figure 3b). The volcano plot highlighted the most significantly differentially expressed genes between the cohorts (Figure 3c).

**Figure 4:**
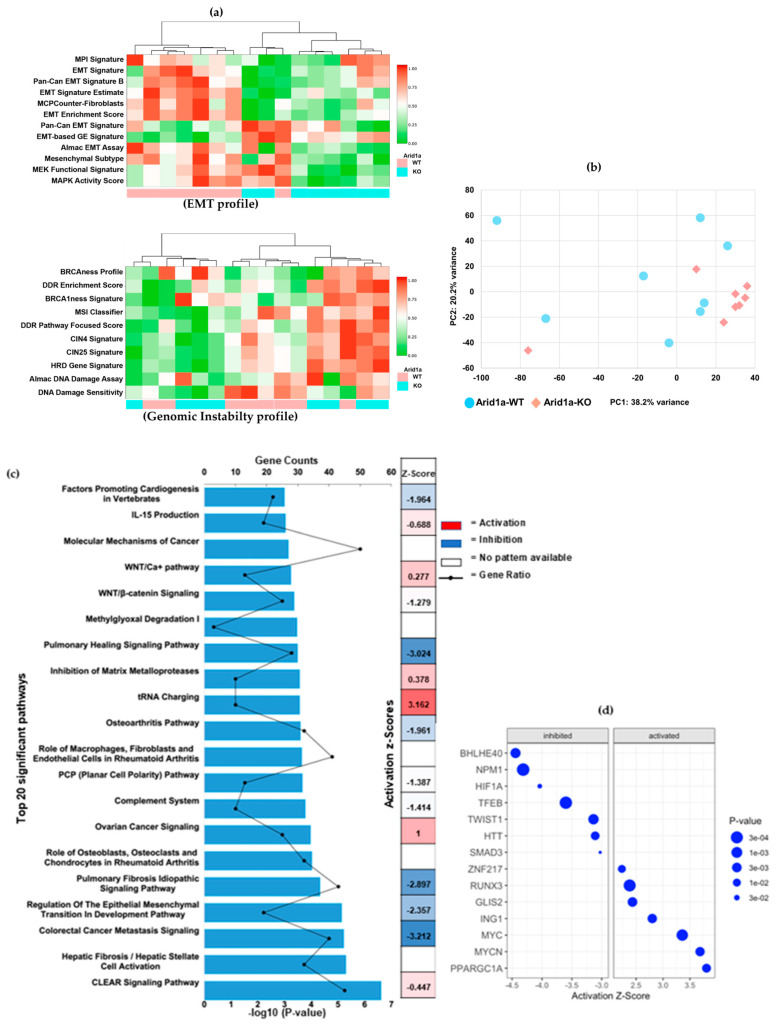
Molecular pathway analysis. (**a**) mRNA profiling by Almac categorized the differential expression profile based on the hallmarks of cancers. Heatmaps showing the topmost significantly correlated hallmarks, (**top**) EMT pathways and (**bottom**) genomic instability. Samples represented by columns and RNA expression signatures represented in rows. Each colored square indicates the expression of the multi-gene signature for that sample (Red = increased expression levels or activation of the hallmark, green = decreased expression levels or repression of the hallmark). (**b**) Principal Component Analysis of the gene expression data. (**c**) IPA analysis showing the top 20 significant signaling pathways correlating the differentially expressed genes between *Arid1a* WT and KO osteosarcomas (*p*-value < 0.05). X-axis representing the −log_10_ *p*-value. (**d**) IPA upstream regulator analysis based on activation Z-score and *p*-values.

To gain further insights into the molecular pathways affected by *Arid1a* loss, we incorporated Ingenuity Pathway Analysis (IPA) and ClaraT mRNA profiling on these differential gene expressions between the two cohorts. ClaraT is an unbiased analysis of DEGs (differentially expressed genes) based on the overall expression patterns of 92 gene signatures across the 10 hallmarks of cancer. For example, in the cohort heatmap in Supplemental Figure S2, the samples are grouped based on their *Arid1a* status into 10 clusters based on correlated gene expression signatures (Spearman rank correlation coefficient ≥ 0.8). Samples were re-clustered independently of their *Arid1a* status, allowing for an unbiased grouping solely based on gene expression similarities and the hallmarks of cancer. ClaraT revealed a correlation in gene expression hallmarks in the genomic instability and EMT regulation pathways that could explain the differences between *Arid1a* WT and KO tumors (Figure 4a). The clustering in Figure 4a demonstrated three similar cohorts even though there were two genetically distinct cohorts based on their *Arid1a* status. Interestingly, the *Arid1a* KO tumors were lower for EMT markers and high for genomic instability. The decrease in mesenchymal markers in the *Arid1a* KO cohort could be correlated to dedifferentiated tumors that are becoming more stem-like. The observed clustering and differential expression patterns highlight the inter- and intratumor heterogeneity between *Arid1a* WT and *Arid1a* KO samples, elucidating the role of *Arid1a* in modulating gene expression. The heterogenous distribution of *Arid1a* knockout samples was also evident from our Principal Component Analysis of RNA-seq data (Figure 4b).

IPA of the DEG identified several enriched canonical pathways. A total of 65 pathways were associated with the DEG (*p*-value < 0.05), and the top 20 pathways with their Z-scores and gene counts are shown in Figure 4c.

Notably, our findings revealed significant inhibitions in pathways associated with differentiation and epithelial-to-mesenchymal-transition (EMT). Intriguingly, the ovarian cancer signaling pathway (*p*-value = 0.0001, Z-score = 1) stood out as prominently activated (Figure 4c). *Arid1a* mutations have been extensively studied in ovarian cancers and are recognized as drivers of oncogenesis, which also explains the activation status of ovarian cancer signaling in our IPA analysis [44]. Supplemental Table S2 provides a comprehensive list of individual genes enriched in each of these pathways.

IPA also revealed 746 enriched upstream regulators including transcription factors, chemical agents, biological drugs, cytokines, kinases, and non-coding RNAs with a *p*-value < 0.05. The top 50 significant upstream regulators and their target molecules in the dataset are summarized in Supplemental Table S3. Notably, the analysis identified *BHLHE40*, *NPM1, HIF1a,* and *TCL1A* as inhibitory transcription regulators and *PPARGC1A*, *MYCN*, *MYC*, *ING1,* and *RUNX3* as activating signals, displaying the highest activation Z-scores and lowest *p*-values (Figure 4d). In addition to these coding genes, upstream regulator analysis identified several miRNAs with a *p*-value < 0.05, including well-known oncogenic miRNAs and tumor suppressor miRNAs such as miR-17, miR-29, miR-32, miR-143, miR-200, and let-7, which have previously been implicated in OS [45,46,47]. A list of these significant miRNAs is provided in Supplemental Table S4.

Using the regulatory effects analysis algorithm within IPA, we investigated the potential connection between the upstream regulators and downstream biology. The regulatory effects that most closely resemble DEG upon *Arid1a* deletion were the activation of myeloid cells, cell movement of myeloid cells, cell movement of phagocytes, inflammatory response, and the phosphorylation of protein. Furthermore, our IPA analysis focusing on disease and cellular function with a threshold of −log_10_ (*p*-value) > 4 revealed the phenotypes wherein the *Arid1a* knockout demonstrated significant resemblance. These included cellular movement, immune-cell trafficking, inflammatory response, organismal death, and developmental disorders. Overall, our transcriptomic data verified the phenotypic changes observed in the animal models of OS, indicating that knocking down *Arid1a* has an impact on preexisting genomic instability leading to more unstable and tumorigenic clusters with a greater potential to metastasize through the loss of mesenchymal markers.

### 3.4. Arid1a Loss Increases Genomic Instability by Disrupting Important Cell Repair Pathways

**Figure 5:**
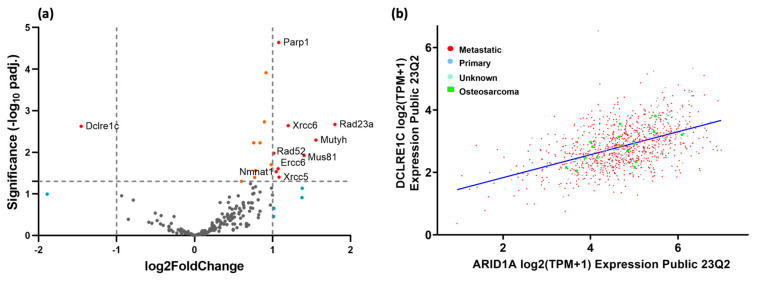
Impact of *Arid1a* loss on DNA damage repair pathways. (**a**) Volcano plot displaying the most significant differentially regulated genes involved in DNA repair pathways between *Arid1a* KO and WT mouse OS; the X-axis represents log_2_ fold change (thresholds of −1 and 1) and the Y-axis shows the adjusted −log10 *p*-values (threshold 0.05). (**b**) Correlation between *DCLRE1C/Artemis* and *Arid1a* expression in all human cancer cell lines (n = 1450) and in OS cell lines (n = 16) from the CCLE database (R^2^ = 0.435, *p*-value = 4.3 × 10^−68^ and R^2^ = 0.581, *p*-value = 0.0183, respectively).

To further investigate the effect of *Arid1a* knockout on the cellular repair mechanisms, we analyzed the differential expression profile of 150 genes involved in the DNA damage repair pathways, using gene set enrichment analysis (GSEA). We observed significant differences in the expression of these genes in the *Arid1a* knockout group. Notably, several genes involved in DNA damage repair pathways exhibited statistically significant upregulation, such as *PARP1* (Poly [ADP-ribose] polymerase 1), *RECQL5* (RecQ protein-like 5), *ATRIP* (ATR interacting protein), *RAD23A* (RAD23 homolog A), *XRCC6* (X-ray repair cross-complementing protein 6, also known as Ku70), *NBN* (Nibrin), *ERCC2* (Excision repair cross-complementation group 2, also known as XPD), *RAD52* (RAD52 homolog), *MUS81* (MUS81 structure-specific endonuclease subunit), *POLL* (DNA polymerase lambda), *MLH1* (MutL homolog 1), *ERCC6* (Excision repair cross-complementation group 6, also known as CSB) and *XRCC5* (X-ray repair cross-complementing protein 5, also known as Ku80) (Figure 5a). These genes are involved in DNA damage arising from both double-strand breaks and single-strand damage, which is likely a result of an activated feedback loop due to the increased DNA damage.

The one gene that directly correlated with *Arid1a* expression was *DCLRE1C* (DNA cross-link repair 1C, also known as Artemis). The log_2_ fold change between cohorts was −1.5 with an adjusted *p*-value of 0.0024 (Figure 5a). The mean raw expression in the *Arid1a* KO cohort was 113.4 ± 51.2 counts, and for the *Arid1a* WT cohort, the mean expression was 684.6 ± 102.2 counts.

Because of this unique correlation among the DNA repair genes, we hypothesized that there might be a broader connection between *Arid1a* and *DCLRE1C*. We, therefore, interrogated extant databases on the expression of these genes and how correlated they are to each other. Upon examination of the CCLE gene expression database for all established cancer cell lines, we found a direct correlation between the two gene expressions. In total, 1450 cancer cell lines were evaluated using a Pearson correlation linear regression analysis where the R^2^ value was 0.435, and the *p*-value was 4.3 × 10^−68^. Looking only at the subset of osteosarcoma cell lines (n = 16), we found a slightly improved correlation coefficient of 0.581 and still a significant *p*-value of 0.0183 (Figure 5b).

### 3.5. In Vitro Disruption of Arid1a Results in More Aggressive Cellular Phenotypes

**Figure 6:**
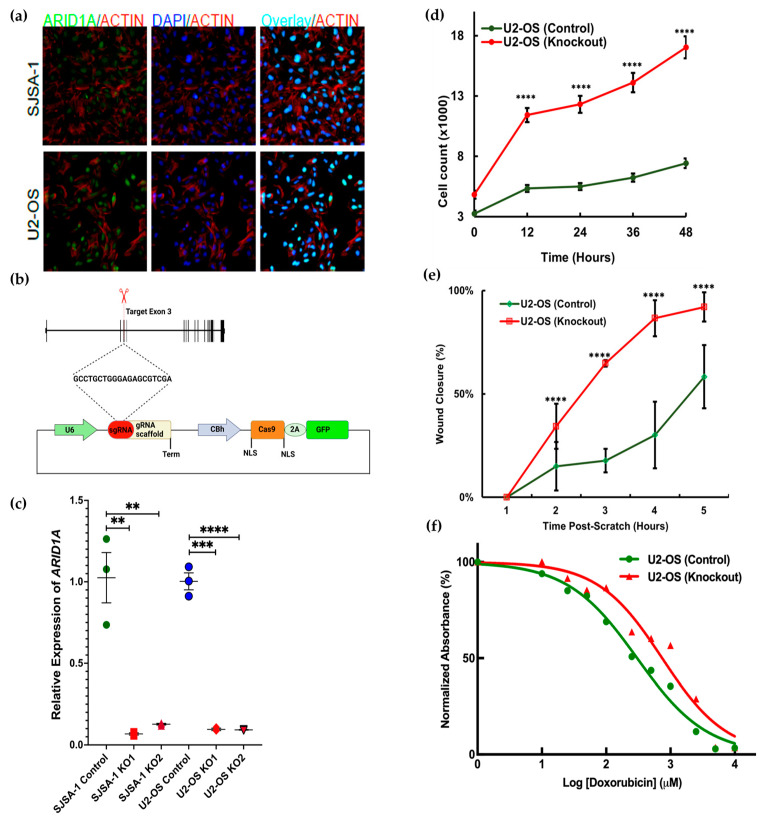
In vitro CRISPR/Cas9 knockout resulted in a more proliferative and chemoresistant cellular phenotype. (**a**) Endogenous expression of *Arid1a* in osteosarcoma cell lines as demonstrated by immunofluorescence staining showing nuclear localization of *Arid1a* (Actin = Red, Nuclei = Blue). (**b**) Schematic for targeting *Arid1a* with CRISPR/Cas9. (**c**) Relative mRNA expression of *Arid1a* after knockout in two biological replicates of U2-OS and SJSA-1. The data represent an average of three technical replicates and error bars represent the standard error of the mean (** *p*-value < 0.05; *** *p*-value < 0.005; **** *p*-value < 0.0005). (**d**) Real-time cell proliferation assay for 48 h in U2-OS cell lines, error bar represents the standard error of the mean (**** *p*-value < 0.0001; individual *t*-tests compared between groups at different time points), (n = 5). (**e**) Real-time cell migration assay at different time points (T1 = 0 h, T5 = 24 h), error bars represents the standard deviation of mean (**** *p*-value < 0.0001), (n = 3). (**f**) Doxorubicin chemosensitivity assay at 72 h, IC_50_ for KO U2-OS = 0.7 μM, and Control U2-OS 0.3 μM.

In vivo studies confirmed that the loss of *Arid1a* leads to more aggressive OS phenotypes, which aligns with the results of the *piggyBac* screen. Previous studies on cell line and tumor tissues have also shown a similar pattern, suggesting that *Arid1a* serves as a tumor suppressor in OS cell lines [27]. The next step was to validate the tumor-suppressive function of *Arid1a* in vitro. U2-OS and SJSA-1 were selected based on their relative expression of *Arid1a* mRNA obtained from the cancer cell line encyclopedia (CCLE) database search (Supplemental Table S5). Endogenous expression was confirmed using RT-qPCR to detect the mRNA (Supplemental Figure S3a) and immunofluorescence detected *Arid1a* protein expression in both cell lines (Figure 6a).

CRISPR/Cas9 was used to alter *Arid1a* in both the U2-OS and SJSA-1 cell lines. Both cell lines were successfully transfected with the *Cas9-GFP* (control) and *Cas9-GFP-Arid1a* knockout plasmids with varied concentrations following the manufacturer’s protocol. The third coding exon of *Arid1a* was selected as the target for guide RNA design (GCCTGCTGGGAGAGCGTCGA) (Figure 6b). High transfection efficiency was detected (>90%) through fluorescent microscopy. A significant reduction in *Arid1a* mRNA expression was detected via RT-qPCR (Figure 6c). Immunoblotting was performed to validate the knockout of *Arid1a*, but without successful detection in the control cell lines.

For phenotypic evaluation, we performed different cell behavior assays and chemosensitivity testing using control and knockout *Arid1a* cell lines. Real-time analysis of cell proliferation on both cell lines using the automated image capture on Image Express Pico revealed that *Arid1a* knockout cells grew markedly faster than the wildtype ones (Figure 6d). We also evaluated cell migratory activity using a scratch migration assay in real-time and noticed a similar trend where *Arid1a* KO cells closed the gap considerably faster than *Arid1a* WT ones (Figure 6e, Supplemental Figure S3b). Furthermore, the MTT survival assay demonstrated that *Arid1a KO* OS cells became more chemoresistant than the *Arid1a WT* cells after 72 h of doxorubicin treatment (ANCOVA, *p*-value < 0.0001, DF = 9, R^2^ = 0.95) (Figure 6f, Supplemental Figure S3c). Overall, in vitro deletion of *Arid1a* led to increased proliferation, migration, and chemoresistance, which correlates with our in vivo findings and the analysis following *Arid1a* loss.

## 4. Discussion

Personalized treatment options for OS face significant obstacles due to the highly complex etiology of the disease, as well as the difficulty in distinguishing between driver and passenger mutations [48,49]. Although genetically engineered mouse models have shed light on the role of *Tp53* and *Rb1* inactivation in osteosarcomagenesis, the results have been highly heterogeneous in terms of finding a consistent driver mutation [13,15,50,51]. In an *SB* genetic screen in mice, Moriarty et al. identified PI3K-mTOR and MYC pathways in OS formation and metastasis [15]. Here, we performed a forward genetic screen using *piggyBac* transposon in a murine model of OS with a background of both *Trp53* and *Rb1* loss. The loss-of-function mutations of both *TP53* and *RB1* are often linked to the development and progression of osteosarcoma [52]. *PB* is an efficient and scarless, cut-and-paste transposon system for mammalian applications. It demonstrates advantages over other transposons by offering a broader genome coverage with a single transposable action, which reduces the noise from clonal expansion of insertion sites [11,51,53,54]. Among the primary tumor samples, we detected 227 integration sites, but very few repeatedly disrupted genes. One of the most frequent integration sites found was in *Arid1a*.

*Arid1a* is one of the cBAF subunits which remodels nucleosomes to open chromatin [16,17]. Mutations in the genes encoding BAF subunits occur in approximately 20% of all human cancers with *Arid1a* being the most frequently mutated [16,18,19,55]. However, the exact mechanisms of how *Arid1a* loss contributes to tumorigenesis vary across tumor types. The primary mechanism is its transcriptional regulation of genes involved in cell differentiation and development, often disrupting enhancer-promoter activity via alterations in histone modifications [18,31,55,56] and DNA methylation [57]. Concomitant loss-of-function mutations in *Arid1a* and *TP53* are observed in several cancers where *Arid1a* enhances the transcriptional activity of P53 [24], thereby suppressing downstream cancer-promoting genes [58]. *Arid1a* mutations often correlate with the DNA damage repair genes enabling synthetic lethality targeting in *Arid1a*-mutant cancers [18,22,34,37]. In endometrial cancer cells with *Arid1a* mutations, increased susceptibility to PARP inhibitors is observed, due to the NHEJ-repair defects caused by the *Arid1a* mutation [37]. Similarly, targeting EZH2 in *Arid1a*-mutated ovarian cancer cells shows synergistic sensitivity [59]. Consequently, many studies using synthetic lethal partners with *Arid1a* deficiency have progressed to clinical trials [22,60,61,62].

The implications of *Arid1a* mutations in OS have only been superficially studied using cancer cell lines. Xu et al. found a strong positive correlation (n = 53, *p*-value < 0.015) between *Arid1a* expression and overall patient survival in OS [27]. A recent clinical study identified a higher rate of *Arid1a* mutations (67%) in patients with worse prognosis [21]. Our in vitro knockout experiments corroborated with these findings, as *Arid1a* loss resulted in a more proliferative and chemoresistant phenotype. Despite that we could not demonstrate the *Arid1a*-KO by western blot, the RT-PCR data and the phenotypic differences indicate that we knocked out *Arid1a* and it was consequential. Together, the body of evidence supports our conclusions. In our in vivo study, we observed efficient osteosarcomagenesis upon deletion of *Arid1a* and a substantial decrease in survival, as well as a dramatic increase in metastatic potential. The premature death of *Arid1a*-deficient mice without visible tumors suggests that either occult tumorigenesis or other factors beyond sarcomagenesis contributed to a reduced lifespan. Interestingly, it appears that biallelic inactivation of *Arid1a* is not necessary to enhance the aggressive phenotype as we observed similar trends of survival and tumor burden between the *Arid1a^fl/fl^* mice and the *Arid1a^fl/wt^* mice. This observation is corroborated by a large pan-cancer study looking at the tumor mutational landscape across 2520 metastatic tumors [63]. Whole genome sequencing revealed that *Arid1a* was one of the most frequently mutated tumor suppressor genes; however, the study also demonstrated that *Arid1a* mutations were least likely to be characterized as a gene deletion, but instead mutations were characterized as INDELS and nonsense mutations and only ~50% of tumors exhibited biallelic inactivation, which is low for a tumor suppressor gene [63].

The enigmatic function of *Arid1a* in cancer has been studied most frequently in ovarian cancer. We observed a remarkable correlation in our gene expression analysis with ovarian cancer signaling (*p*-value = 0.0001, Z-score = 1). *Arid1a* is a commonly mutated gene in this cancer type, with a reported mutation frequency ranging from 30% to 50%. The functional consequences of *Arid1a* loss in ovarian cancer are multifaceted. *Arid1a*-deficient ovarian cancer cells have been shown to exhibit altered gene expression patterns, dysregulated cell cycle progression, impaired DNA damage repair, and an enhanced EMT phenotype. However, decreased expression or *Arid1a* mutational status did not impact overall survival in patients with various types of ovarian cancer [29]. Hence, more needs to be studied not only on the mutational status of *Arid1a* in osteosarcoma, but also the epigenetic mechanisms that regulate gene and protein expression.

Bioinformatic platforms demonstrated increased heterogeneity through hierarchical clustering in the *Arid1a* KO tumors. Tumor cells undergo transitions in their stemness leading to tumor heterogeneity and cellular plasticity. The correlation between cancer stem cells (CSC) and metastasis is ongoing, but studies conducted in OS have identified that CSC markers are associated with metastasis and a worse prognosis [47,64]. In a liver cancer model, *Arid1a* deficiency contributed to an increased number of stem/progenitor-like cells by dysregulating genes related to stemness [65]. Similar consequences were observed in other cancers where *Arid1a* loss increased stemness-associated genes [66,67,68]. The decrease in mesenchymal markers in a mesenchymal tumor highlights dedifferentiation that leads to cancer stemness. Many other pathways of differentiation were downregulated in *Arid1a*-deficient mice. Early studies of EMT were not a proper characterization of the transition from epithelial to mesenchymal, but a loss of epithelial markers. Thus, many of the early EMT algorithms also can be considered analyses of dedifferentiation.

Despite the apparent intertumoral heterogeneity, transcriptomic profiling of OS tumors highlighted molecular mechanisms responsible for an aggressive phenotype. The two cancer hallmarks that explained the gene expression differences between *Arid1a^fl/fl^* and *Arid1a^wt/wt^* were genomic instability and EMT. Genome-wide association studies in metastatic OS using high-throughput sequencing [14] support the correlation between increased genomic instability and mutational burden [7]. Partial or complete *Arid1a* loss has previously been linked to impaired DDR [37]. These studies have focused on variant alleles associated with DNA damage response and repair. In our study, we have observed that the loss of *Arid1a* led to significant upregulation in the expression of mRNAs that are essential for maintaining genome stability, presumably as a feedback mechanism to combat extensive DNA damage. Many of these mRNAs are involved in critical mechanisms for DDR response and most of which were previously been implicated in OS [69,70,71]. There was one gene that directly correlated with decreased expression of *Arid1a,* namely *Dclre1c*. *Dclre1c* is a gene that encodes for the protein Artemis, which is involved in non-homologous end joining often employed to correct double strand breaks in DNA [72]. In a study by Lavi et al., it was also noted that increased *Dclre1c* expression correlated with an improved survival outcome. Out of 48 DNA repair genes, this was the only gene that positively correlated with progression [73]. If the positive expression of *Dclre1c* promotes survival, the antithesis is also true that negative expression is associated with poor survival. Out of 150 DDR genes in our study, *Dclre1c* was the only one to directly correlate with *Arid1a* status and a mouse model with a lower survival.

In addition to the coding regions of the genome, IPA revealed several non-coding transcripts including some recognized oncogenic and tumor suppressor miRNAs [8,45,46,47,74]. Non-coding variants can alter epigenetic states in cancer, thereby regulating oncogenic or tumor suppressive properties of their target genes [75]. Impact of non-coding regions in maintaining DNA integrity and their correlation to chemoresistance and metastasis in cancers is well investigated [8,76]. Functional studies in OS have identified numerous non-coding transcripts [8,74,75,77,78] and potential correlations between genomic stability and ncRNAs [8,45,47]. Studies also demonstrated the role of ncRNAs regulating *Arid1a* expression and their involvement in different cancers [55,79]. We believe that *Arid1a* loss significantly impacts both the coding and non-coding regions of the genome. Therefore, further investigation is needed to functionally characterize these novel non-coding variants that are associated with aggressiveness in *Arid1a*-mutated OS.

## 5. Conclusions

This study establishes that *Arid1a* indeed plays a tumor suppressor role in aggressive osteosarcoma and opens an exciting new avenue for further studies into the molecular mechanisms underlying its effects. The initial genetic screening has laid the groundwork for subsequent validation and molecular characterization, culminating in the identification of precise molecular signatures linked to aggressive OS. Our well-established Cre-mediated, triple-mutation GEMM model offers a robust preclinical platform that closely mimics human OS. This model helps us to understand the molecular changes that occur at various stages, enhancing our understanding of the role of *Arid1a* loss in osteosarcomagenesis. Transcriptomic data and the unbiased analysis of the differentially expressed genes revealed a probable connection between *Arid1a* loss and genomic instability. Although the functional mechanism remains to be characterized, our findings suggest that *Arid1a* plays a crucial role in maintaining genomic integrity. These significant correlations highlight the importance of further investigating the effects of *Arid1a* loss to gain a better understanding of the underlying mechanisms involved. By utilizing our model, we can contribute to the understanding of the specific molecular events and pathways involved in chemoresistance and metastasis in osteosarcoma, particularly focusing on the potential for simultaneous targeting of DNA damage repair proteins in *Arid1a*-mutated osteosarcoma.

## Data Availability

RNA-seq data is available through the GEO database using the accession number GSE247731.

## Supplementary Materials

The following supporting information can be downloaded at: https://www.mdpi.com/article/10.3390/cancers16152725/s1, Figure S1: Schematic of *PBonc* design; Figure S2: Hierarchal clustering of the hallmarks of cancer heatmap between *Arid1a WT* and *Arid1a KO* mice tumors; Figure S3: Impact of in vitro *Arid1a* deletion in cellular phenotypes; Table S1: *piggyBac* insertion sites; Table S2: Ingenuity pathway analysis for gene expression differences; Table S3: Cellular process analysis for gene expression differences; Table S4: miRNA analysis from RNA-seq data; Table S5: *Arid1a* CCLE expression data.
