# Supplementary material for: Arid1a Loss Enhances Disease Progression in a Murine Model of Osteosarcoma
Source: Cancers (Basel). 2024 Jul 31;16(15):2725. doi: 10.3390/cancers16152725 (PMC11311538; doi:10.3390/cancers16152725)
Supplement: Supplementary file 1 [file cancers-16-02725-s001.zip › Supplemental figures.pdf]

Arid1a Loss Enhances Disease Progression in a Murine Model of Osteosarcoma

Kaniz Fatema, Yanliang Wang, Adriene Pavek, Zachary Larson, Christopher Nartker, Shawn Plyler, Amanda Jeppesen, Breanna Mehling, Mario R. Capecchi, Kevin B. Jones and Jared J. Barrott

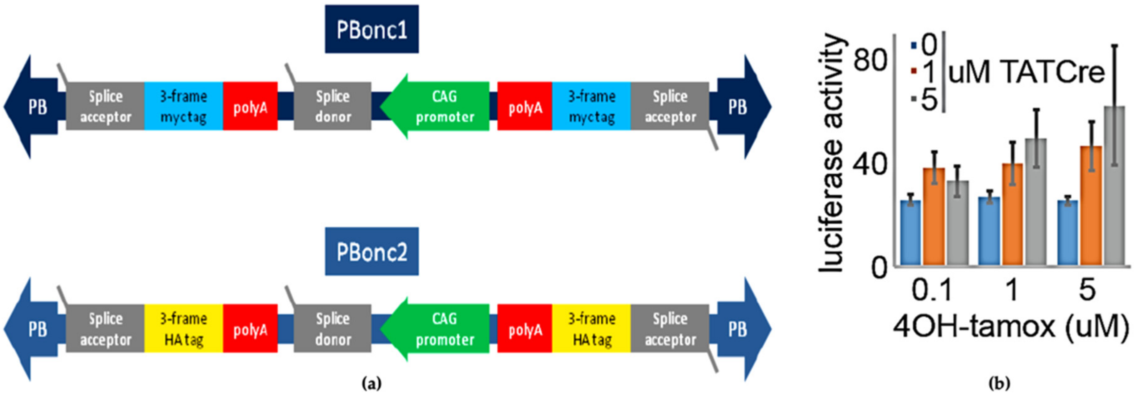

(a) (b)

Supplemental Figure S1. Schematic of *PBonc* design. (**a**) Schematic of transposable elements design, PBonc1 and PBonc2, each including birectional splice acceptors followed by triple-frame epitope tags and poly-adenylation signals as well as a strong promoter and splice donor site in the middle of each; (**b**) Luciferase activity showing Cre-and tamoxifen dependency for the PBonc fibroblasts.

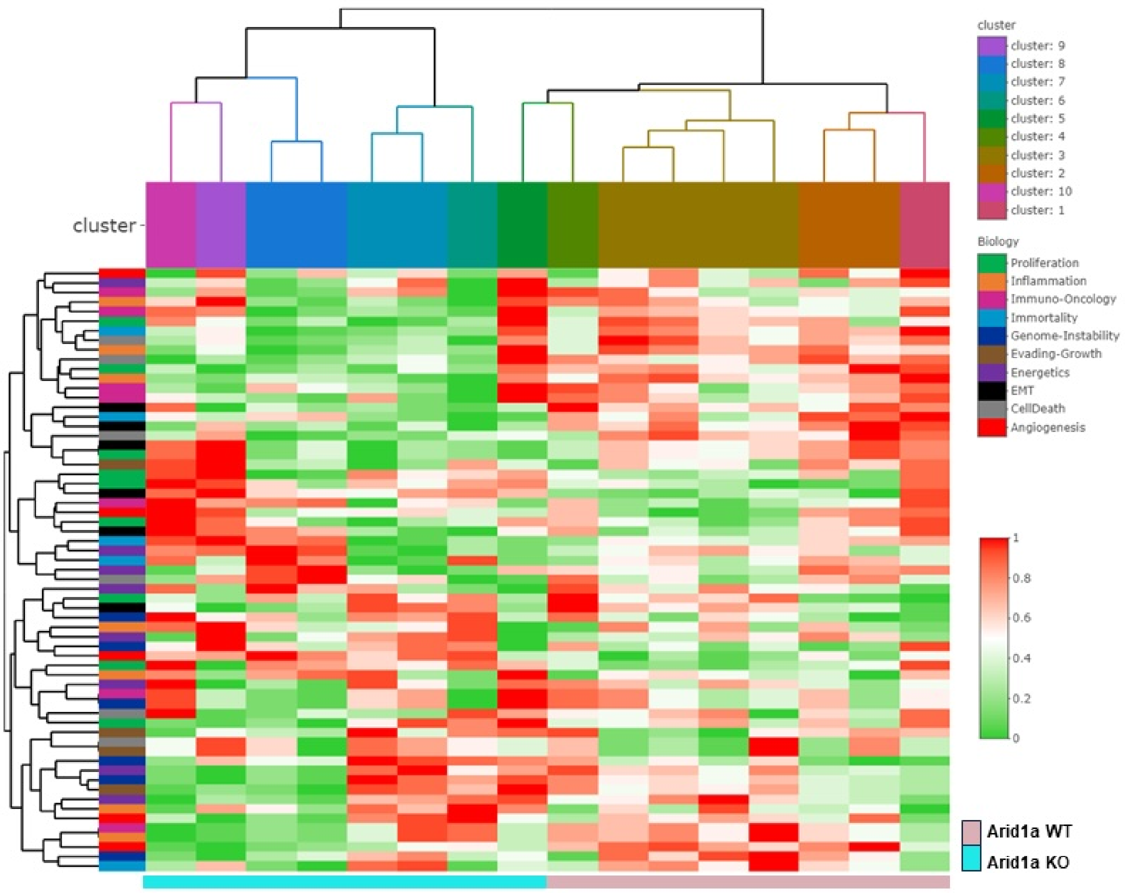

Supplemental Figure S2. Hierarchal clustering of the hallmarks of cancer heatmap between *Arid1a WT* and *Arid1a KO* mice tumors. The hallmarks of cancer are indicated by colored bars on the left. Mouse genotypes are indicated by the teal (*Arid1a KO*) and pink (*Arid1a WT*) colors across the bottom.

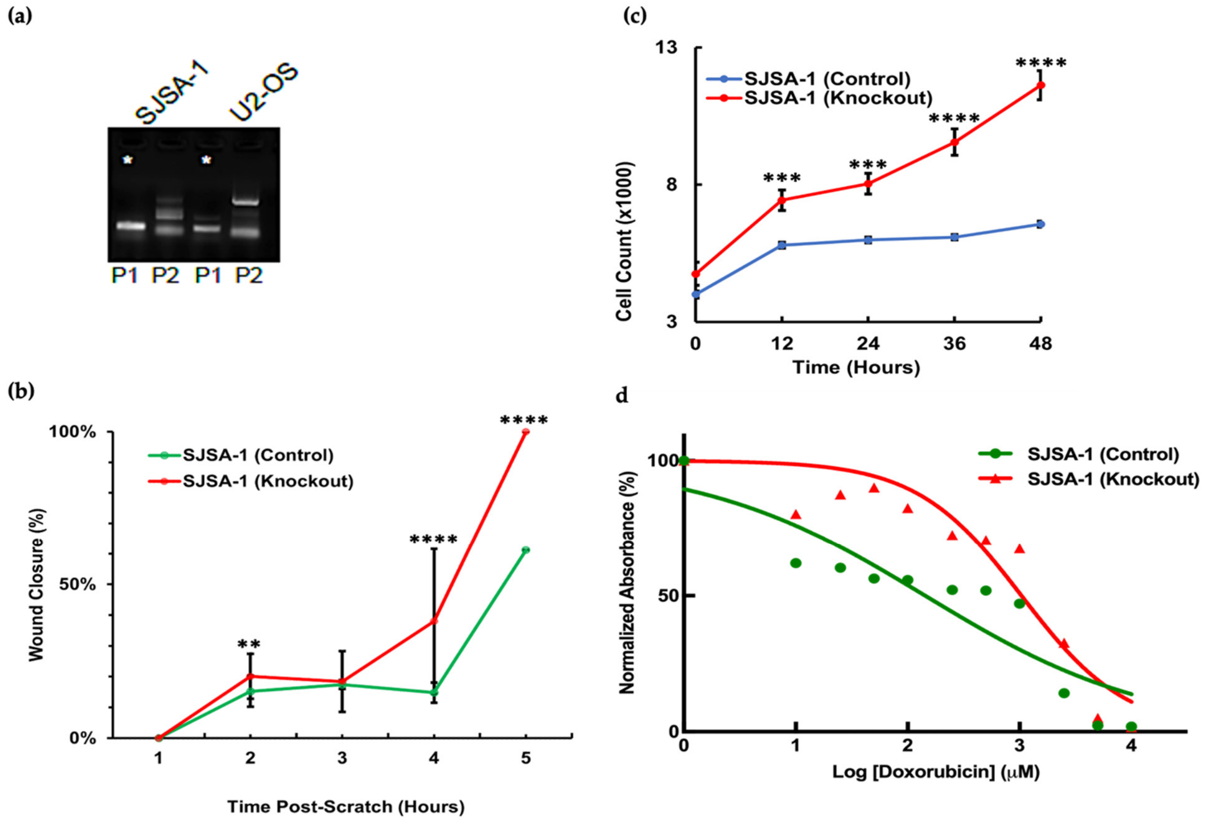

Supplemental Figure S3. Impact of *In vitro ARID1A* deletion in cellular phenotypes. (**a**) *ARID1A* (RT-qPCR) mRNA detection using different primer sets in osteosarcoma cell lines; (**b**) Real-time cell proliferation assay for 48 hours in SJSA-1 cell lines, error bar represents the standard error of the mean (** p-value < 0.01; **** p-value < 0.0001; individual t-tests compared between groups at different time points), (n = 5); (**c**) Real-time cell migration assay at different time points (T1 = 0 hours, T5 = 24 hours), error bars represents the standard deviation (*** p-value < 0.001; **** p-value < 0.0001), (n = 3); (**d**) Doxorubicin chemosensitivity assay at 72 hours, IC_{50} for KO SJSA-1 = 1.0 μM, and Control SJSA-1 = 0.1 μM, (n = 7).
